# Therapeutic potential of acidic cannabinoids: an update

**DOI:** 10.1186/s42238-026-00387-y

**Published:** 2026-01-16

**Authors:** Santosh Kumar Singh, Coralie Antoine, Calvin Tse, Lawrence Ji, Miranda Reed, Wayne Grant Carter, Viviana Trezza, Hemant Kumar Bid

**Affiliations:** 1 Department of Microbiology, Biochemistry, and Immunology, Office of Online Education and Expanded Programs (OEEP), Morehouse School of Medicine, Morehouse School of Medicine, 720 Westview Drive SW, Atlanta, GA 30310 USA; 2 Essentia Scientific, San Leandro, CA 94577 USA; 3 Harrison College of Pharmacy, Auburn University, Auburn, AL 36849 USA; 4 School of Medicine Education Centre, University of Nottingham, Royal Derby Hospital Centre, Derby, DE22 3DT UK; 5 Department of Science, Roma Tre University, Viale Marconi 446, Rome, 00146 Italy

**Keywords:** Tetrahydrocannabinolic acid (THCA), Cannabichromenic acid (CBCA), Cannabigerolic acid (CBGA), And cannabidiolic acid (CBDA), Cannabidiol (CBD), Livetolic acid (OLA), Cancer, Epilepsy, Alzheimer, Pain, Inflammation

## Abstract

Cannabis sativa yields a wide range of bioactive compounds, including terpenes, flavonoids, and cannabinoids. Tetrahydrocannabinolic acid (THCA), cannabidiolic acid (CBDA), cannabigerolic acid (CBGA), and cannabichromenic acid (CBCA) are the acidic biosynthetic precursors of the neutral cannabinoids Δ9-tetrahydrocannabinol (THC) and cannabidiol (CBD), which have been the subject of much research. This review examines the biosynthesis, decarboxylation, molecular pharmacology, and therapeutic significance of acidic cannabinoids, intending to address a significant knowledge gap. Peer-reviewed literature from major scientific databases was used in a systematic narrative review with an emphasis on investigations of acidic cannabinoid chemistry, pharmacology, pharmacokinetics, and disease-specific applications. According to the reviewed data, acidic cannabinoids exhibit unique biological activities that distinguish them from their neutral counterparts. These include neuroprotective, anti-inflammatory, anticonvulsant, and anti-proliferative actions, which are mediated by molecular targets such as serotonin 5-HT1A receptors, cyclooxygenase-2 (COX-2), transient receptor potential (TRP) channels, and peroxisome proliferator-activated receptor-γ (PPARγ). Acidic cannabinoids are more appealing for therapeutic usage in children and the elderly, considering that they are not intoxicating like THC; however, this distinction applies primarily to non‑heated consumption. Chemical instability, low bioavailability, and a dearth of controlled human trials impede clinical translation despite their potential. According to the findings, acidic cannabinoids are an underutilized yet potentially valuable class of precision medicines. In this study, we outline existing understanding on acidic cannabinoids, discuss their production and transformation, and identify research needs that could influence cannabis science research.

## Introduction

Comprised of endogenous ligands, cannabinoid receptors, and enzymes for their synthesis and breakdown, the endocannabinoid system (ECS) is a crucial regulator of neuronal activity, network function, and immunological responses. CB1 receptors, predominantly located in the central nervous system, mediate effects on cognition, memory, pain perception, and motor control, while CB2 receptors, mainly found on immune cells and microglia, regulate inflammatory processes (Gamage and Lichtman 2012). The principal endocannabinoids—2-arachidonoylglycerol (2-AG) and anandamide (AEA)—are synthesized from membrane phospholipids via phospholipase C (PLC), diacylglycerol lipase (DAGL), and other specialized enzymes, and are inactivated by fatty acid amide hydrolase (FAAH) or monoacylglycerol lipase (MAGL). Phytocannabinoids from *Cannabis sativa* modulates this system, with Δ9-tetrahydrocannabinol (THC) acting as a potent CB1 and CB2 agonist responsible for psychoactive and psychotomimetic effects (D'Souza et al. 2008), while cannabidiol (CBD) primarily targets serotonin 5-HT1A receptors, Transient Receptor Potential Vanilloid 1 (TRPV1), and GPR55, exerting anti-inflammatory, anxiolytic, and antiepileptic properties (Burstein 2015; De Petrocellis and Di Marzo 2010).

CB1 receptors are found in the frontal cortex and spinal cord, among other parts of the central nervous system, and CB2 receptors are found primarily in the immune system and peripheral tissues, where they are markedly upregulated in response to pathological conditions (Chayasirisobhon 2020). While both AEA and 2-AG have a high propensity for binding to the CB1 and CB2 receptors, AEA has the highest affinity. As a result, membrane phospholipid precursors are cleaved in response to physiological or pathological stimuli, these endocannabinoids are synthesized and released quickly, demonstrating their function in "on-demand" signaling (Chayasirisobhon 2020). The G protein–coupled CB1 and CB2 receptors are targets of this cannabinoids, with CBD possibly functioning as a partial agonist at the CB2 receptor (Castillo-Arellano et al. 2023). CBD may reduce symptoms such as pain, inflammation, oxidative stress, and degeneration of neuronal excitability through its interaction with the endocannabinoid system (ECS) (Castillo-Arellano et al. 2023; Martinez Naya, et al. 2023). It could additionally preserve glial cell homeostasis and prevent the advancement of cancer and neurodegenerative diseases. When 10 mg of CBD is administered, the highest plasma concentration of approximately 3 μg/L is reached after about 2.8 h, despite its low affinity for cannabinoid receptors at clinical dosages (Castillo-Arellano et al. 2023). There is considerable ambiguity about the role of CB receptor activity in the anticonvulsant effects of CBD, given that it may function as a negative allosteric modulator at the CB1 receptor. The antiseizure effects of CBD have also been attributed to its antagonistic effects on transient receptor potential cation channels, including TRPA1 and TRPV1-4, as well as orphan G-protein receptors like GPR18 and GPR55. Additionally, CBD is an antagonist of the µ and δ opioid receptors and an inverse agonist of the GPR3, GPR6, and GPR12 receptors. Its control of intracellular calcium (Ca2 +) levels by blocking T-type voltage-gated calcium channels and antagonizing GPR55, as well as its function as a full agonist at vanilloid channels TRPA1 and TRPV1-4, is probably primarily responsible for its antiseizure efficacy (Borowicz-Reutt et al. 2024).

While most research has focused on neutral cannabinoids such as THC and CBD, increasing attention is being given to their non-psychoactive acidic precursors—tetrahydrocannabinolic acid (THCA), cannabidiolic acid (CBDA), and cannabigerolic acid (CBGA). These compounds, synthesized from CBGA via THCA synthase and CBDA synthase, display unique pharmacological actions, including cyclooxygenase-2 (COX-2) inhibition, peroxisome proliferator-activated receptor gamma (PPARγ) activation, transient receptor potential (TRP) channel modulation, and serotonin 5-HT1A receptor activity (Li et al. 2024; Russo 2011). Preclinical studies suggest that CBDA and THCA possess anti-inflammatory, anticonvulsant, neuroprotective, anti-nausea, and anti-cancer properties without the intoxicating effects of THC, making them promising candidates for long-term therapy in both pediatric and geriatric populations. However, challenges such as chemical instability, limited bioavailability, and a scarcity of clinical trials currently restrict their integration into mainstream medicine, underscoring the need for further pharmacological, clinical, and formulation research. Table 1 shows an analysis of major phytocannabinoids, including THC, CBD, CBG, CBN, CBDA, CBGA, and THCA. It outlines their molecular targets, relevance to pathological conditions, and pharmacological actions. These data are designed to support translational research, therapeutic development, and clinical applications of cannabinoid-based medicine. The present reviews explores the existing understanding on acidic cannabinoids, discusses their transformation, and identifies the research needs that could influence cannabis science research.

**Table 1:** Major Phytocannabinoids, their molecular targets, pathological conditions affected, and pharmacological effects

Cannabinoid	Primary Receptors	Associated Diseases	Therapeutic Effects	Known Inhibitors/Modifiers	References
THC	CB1, CB2	Chronic pain, nausea, PTSD, multiple sclerosis	Analgesic, anti-nausea, appetite stimulation, psychotomimetic	Rimonabant (CB1 inverse agonist)	D'Souza et al. 2008; Meanti et al. 2025; Haney et al. 2023)
CBD	5-HT1A, TRPV1, GPR55	Epilepsy, anxiety, inflammation, schizophrenia	Anxiolytic, anti-inflammatory, antipsychotic	Capsazepine (TRPV1 antagonist)	Storozhuk 2023; Kovalchuk and Kovalchuk 2020)
CBN	CB2 (weak), TRPA1	Sleep disorders, pain, glaucoma	Sedative, analgesic, anti-inflammatory	HC-030031 (TRPA1 inhibitor)	Kovalchuk and Kovalchuk 2020; Khouchlaa et al. 2024)
CBG	CB1 (partial), α2-adrenergic, TRPM8	IBD, neurodegeneration, bacterial infections	Anti-inflammatory, neuroprotective, antibacterial	AM251 (CB1 antagonist), Yohimbine (α2-antagonist)	Kovalchuk and Kovalchuk 2020; Krzyzewska et al. 2025)
CBDA	COX-2, TRPV1	Breast cancer, inflammation, nausea	Anti-proliferative, anti-inflammatory	Celecoxib (COX-2 inhibitor)	Takeda et al. 2008; Anderson et al. 2019)
CBGA	PPARγ, TRPA1, TRPV1	Alzheimer’s disease, oxidative stress, neuroinflammation, AKI	Neuroprotective, antioxidant, anti-inflammatory, antitumoral, appetite-stimulating	GW9662 (PPARγ antagonist) TRPM7	Kovalchuk and Kovalchuk 2020; Tusl 1972; Nadal et al. 2017; Navarro et al. 2018; Suzuki et al. 2023)
CBCA	CB1, CB2, TRPA1, TRPV1, 5-HT3, Glycine, Glycine α2	N/A	Antibacterial, anti-seizure	N/A	Khouchlaa et al. 2024; Sepulveda et al. 2024; Lacerda et al. 2025)
THCA	CB1 (inactive), COX-2, TRPA1	Prostate cancer, inflammation	Anti-inflammatory, anti-proliferative	NS-398 (COX-2 inhibitor)	Khouchlaa et al. 2024; Anderson et al. 2019; Nadal et al. 2017; Palomares 2023)

## Methods

This review adopted a narrative and integrative literature review approach to synthesize current findings on the pharmacology, biosynthesis, and therapeutic potential of acidic cannabinoids—specifically THCA, CBDA, and CBGA—in disease contexts such as cancer, pain, epilepsy, and neurodegenerative disorders. The primary goal of this study was to critically analyze and compile recent data on the molecular mechanisms, receptor interactions, and pharmacokinetics of these acidic cannabinoids, to identify research gaps and translational obstacles. Systematic searches of electronic databases, including PubMed, ScienceDirect, Scopus, and Google Scholar, were conducted to identify relevant studies. Data were compared across computational, in vitro, and in vivo models whenever feasible. Pharmacology, biosynthesis, neuroprotection, anti-inflammatory, and anti-cancer pathways were used to categorize the studies.

### Biosynthesis and decarboxylation of acidic cannabinoids

**Fig. 1:**
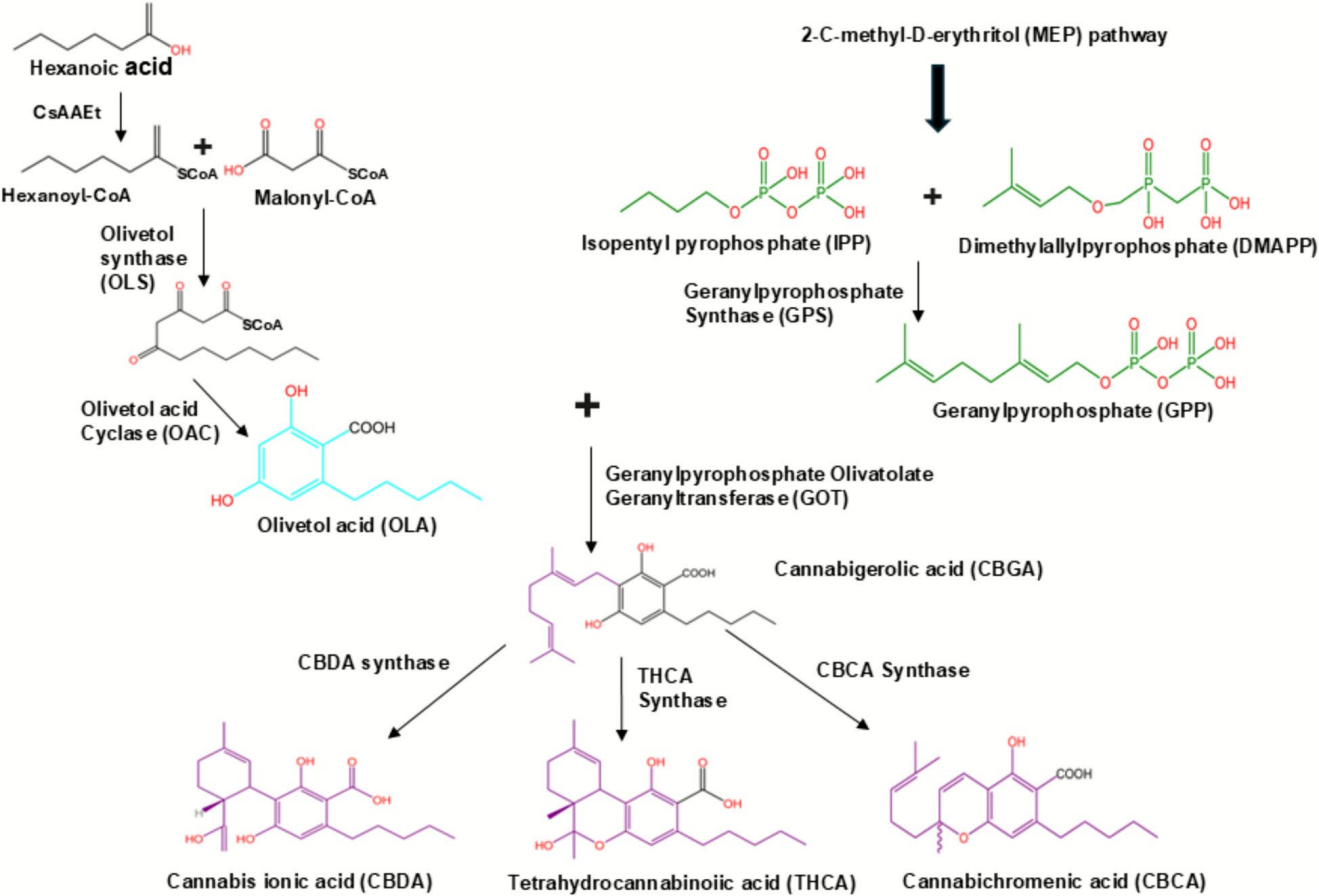
Illustrates the biosynthesis of CBDA, THCA, and CBCA

The molecular level of cannabinoids biosynthesis is still under investigation. They share a common initial pathway, involving tetraketide synthase (TKS), a type III polyketide synthase. The process involves sequential condensation of hexanoyl-CoA with malonyl-CoA, resulting in 3,5,7-trioxododecaneoyl-CoA. This is then cyclized and aromatized by olivetolic acid cyclase (OAC) to olivetolic acid (OLA) (Tahir et al. 2021**)**. The biosynthesis of acidic cannabinoids in *Cannabis sativa* begins in the glandular trichomes and follows a highly coordinated interaction between the polyketide pathway and the methylerythritol phosphate (MEP) pathway (Tahir et al. 2021). In the polyketide pathway, fatty acid metabolism produces hexanoyl-CoA, which is condensed with three molecules of malonyl-CoA by the type III polyketide synthase tetraketide synthase (TKS) to form a tetraketide intermediate. This unstable intermediate is then cyclized and aromatized by OAC into OLA, a resorcinolic compound that forms the aromatic core of cannabinoids (Tahir et al. 2021; Gagne et al. 2012). In parallel, the MEP pathway—localized in plastids—generates geranyl pyrophosphate (GPP) via geranyl pyrophosphate synthase (GPPS). The convergence of these two pathways occurs through a prenylation reaction catalyzed by geranyl olivetolate transferase (GOT), also called CBGA synthase, where GPP is transferred to OLA to form CBGA (Gulck and Moller 2020). CBGA is widely referred to as the “mother cannabinoid” because it serves as the central biochemical precursor for THCA, CBDA, and cannabichromenic acid (CBCA). These conversions are mediated by FAD-dependent oxidocyclase enzymes—THCA synthase, CBDA synthase, and CBCA synthase—whose expression patterns vary by plant genotype, environmental factors, and developmental stage (Tahir et al. 2021; Flores-Sanchez and Verpoorte 2008). The biosynthesis of CBDA, THCA, and CBCA are shown in Fig. 1.

The decarboxylation of acidic cannabinoids is a non-enzymatic thermal process in which the carboxyl group (–COOH) is cleaved as carbon dioxide (CO₂), yielding neutral cannabinoids with enhanced pharmacological activity. For example, THCA decarboxylates to psychoactive THC, CBDA to CBD, and CBGA to CBG (Wang et al. 2016). Decarboxylation can also occur slowly at ambient temperature under prolonged storage, although heat is the dominant driver during processing methods such as smoking, vaping, and baking (Tahir et al. 2021). The reaction is temperature- and time-dependent: optimal conversion for THCA typically occurs between 105–145°Cover several minutes, while excessive heating accelerates oxidative degradation to byproducts such as cannabinol (CBN). CBDA and CBGA exhibit similar thermal behavior but with subtle differences in activation energies, which may influence their ideal processing parameters (Perrotin-Brunel et al. 2010). Kinetically, THCA decarboxylation has been modeled as an acid-catalyzed, pseudo–first-order reaction involving keto–enol tautomerization before CO₂ release (Perrotin-Brunel et al. 2010). Analytical advances, particularly ultra-high-performance supercritical fluid chromatography (UHPSFC) coupled with photodiode array–mass spectrometry, have allowed researchers to monitor acidic and neutral cannabinoids simultaneously without inducing artificial decarboxylation during analysis (Wang et al. 2016; Citti et al. 2019).

In *Cannabis sativa*, the enzyme cannabichromenic-acid synthase (CBCAs), which is dependent on FAD, transforms CBGA into the bicyclic structure of CBCA (Tahir et al. 2021; Thomas and Kayser 2023; Morimoto et al. 1998). This enzyme was initially identified from cannabis flowers, confirming its function in the phytocannabinoid pathway in conjunction with THCA- and CBDA-synthases (Fulvio et al. 2023). One of the first cannabinoids to be created during plant growth is CBCA, and its levels correspond to the expression of the CBCA_S_ gene. Recent bioengineering attempts have concentrated on improving the performance of CBCA_S_ through logical protein design and reestablishing the CBCA pathway in yeast due to limits in its natural function (Tahir et al. 2021; Thomas et al. 2020; Luo et al. 2019; Qi et al. 2025). These techniques have provided efficient ways to produce CBCA at research-level scales and enhanced CBCA yields from glucose-derived sources.

Understanding these biosynthetic and decarboxylation processes is essential for both pharmaceutical formulation and industrial cannabis production. In cultivation, optimizing light intensity, temperature, and nutrient availability can modulate CBGA production and downstream synthase activity to favor desired cannabinoid profiles (Suzuki et al. 2023; Tahir et al. 2021; Urvashi et al. 2024; Reichel et al. 2022; Park et al. 2022). In processing, precise decarboxylation protocols can maximize therapeutic potency while preserving volatile terpenes and preventing oxidative degradation. Beyond plant-based production, recent metabolic engineering approaches have replicated key biosynthetic enzymes in yeast and bacteria, enabling microbial cannabinoid biosynthesis for large-scale production of rare acidic cannabinoids without agricultural variability (Flores-Sanchez and Verpoorte 2008; Thomas et al. 2020; Luo et al. 2019). As clinical research expands into both neutral and acidic cannabinoids—given the latter’s unique anti-inflammatory, neuroprotective, and antiemetic properties—refined control of biosynthesis and decarboxylation will be pivotal to unlocking their full therapeutic potential.

### Pharmacology of acidic cannabinoids

Even while studies on the medicinal potential of Phytocannabinoid acids are growing, little is known about these ubiquitous substances' fundamental pharmacodynamic and pharmacokinetic properties. Given their anti-inflammatory, anti-hyperalgesic, and anxiolytic properties in animal models, CBDA and THCA may find extensive use in medicine. THCA seems to be the primary factor driving the anti-inflammatory properties of Cannabis sativa extracts in cellular models of inflammatory bowel illness, and they also show antiemetic effects in rats and shrews (Rock et al. 2013; Nallathambi et al. 2017).

Because they are found in large quantities in artisanal Cannabis sativa oils used to treat uncontrollable juvenile epilepsies, CBDA and THCA have been hypothesized to have anticonvulsant qualities. Too far, however, no study has reported this molecule's anticonvulsant properties in the peer-reviewed scientific literature. Finding new therapeutic uses and determining the best time to dose for studies using in vivo illness models depend heavily on collecting and sharing phytocannabinoid acid pharmacokinetic data.

Through enzymatic mechanisms involving CBDA synthase and THCA synthase, respectively, the Cannabis plant produces both CBDA and THCA. The two main types of cannabinoids found in raw Cannabis, CBDA and THCA, are produced by these enzymes from CBGA (Tahir et al. 2021; Gulck and Moller 2020; Morimoto et al. 1998). These acidic cannabinoids decarboxylate when exposed to heat or light, changing into their neutral forms, THC and CBD, which are more extensively researched for their psychotropic and medicinal properties. Both CBDA and THCA, in contrast to THC, are non-psychoactive, which means they do not result in the "high" that comes with using Cannabis (McPartland et al. 2015; Pertwee 2008). CBDA may have anti-inflammatory qualities because it has been found to inhibit the inflammatory enzyme COX-2 selectively. Furthermore, CBDA shows an affinity for serotonin 5-HT1A receptors, which could be a factor in its possible anti-anxiety and anti-nausea properties (Bolognini et al. 2013). However, THCA has shown anti-inflammatory and neuroprotective qualities, which may be due to its suppression of pro-inflammatory enzymes and interaction with PPARγ (Nadal et al. 2017).

According to research on THCA and CBDA's pharmacokinetics, these substances are quickly absorbed when taken orally, reaching plasma peak concentrations (Tmax) in 15 to 45 min (Anderson et al. 2019). However, under normal circumstances, they appear to have limited penetration into the central nervous system due to their low brain-to-plasma ratios and relatively short half-lives (less than 4 h). Notably, the brain-plasma ratio of CBDA increases dramatically when taken via a Tween 80-based vehicle, suggesting that formulation strategies can affect the drug's distribution and bioavailability**.** The CBC, CBD, and CBG acidic forms are CBCA, CBDA, and CBGA, respectively. Compared to their neutral versions, their pharmacokinetic characteristics and metabolism have received less research attention. Heat is used to change the acidic forms into their neutral ones, but it is crucial to know if this happens in vivo. Although CBCA and CBGA in their decarboxylated forms did not convert in mice, CBD was found in plasma at 0.5% of the concentration of CBDA after administration, indicating that CBDA might have been converted to CBD in vivo (Anderson et al. 2019).

*Cannabis sativa*'s CBCA synthase produces CBCA, an acidic phytocannabinoid which is understudied. It is the precursor of cannabichromene (CBC) and has attracted attention given its effects on ABC (ATP-binding cassette) transporters, especially as an ABCB1 substrate (Colvin et al. 2022), which may have impacts on drug bioavailability and absorption. The direct effects of CBCA are still mostly unknown, even though most pharmacological insights are derived from CBC, which has anti-inflammatory, analgesic, and possibly anticonvulsant qualities. According to plant latitudinal origin, CBCA is often the third most abundant biogenetic phytocannabinoid in the Cannabis plant, while it is frequently more numerous than CBDA in tropical specimens (Amaral Silva et al. 2020). The plant produces CBC (Etchart et al. 2022) through the non-enzymatic decarboxylation of CBCA, which is synthesized enzymatically. According to preclinical studies, CBC and CBCA both have promising therapeutic uses. A mouse model of intractable infantile epilepsy showed that CBCA was anticonvulsant. Lastly, it has been observed that CBCA exhibits antibacterial qualities against MRSA, or methicillin-resistant Staphylococcus aureus (Lacerda et al. 2025). 

A clinical study on CBCA revealed a high peak plasma concentration (Cmax = 8.4 ± 1.4 μg/mL) 30 min after an intraperitoneal administration of 10 mg/kg. It took several hours to distribute into brain tissue, reaching tmax at 60 min. In the brain, CBCA had a longer half-life (t1/2 = 136 min) than in plasma (Anderson et al. 2021), which was 35 min. Despite having a lower overall drug exposure in brain tissue than in plasma, CBCA demonstrated a notable amount of brain penetration (brain–plasma ratio of 0.53). This significant CBCA brain penetration contrasts with earlier investigations, in which CBCA was not found in brain tissue after an intraperitoneal dose of 5 mg/kg. Administering medications with nonionic surfactants, such as Tween80, has been shown to increase biomembrane permeability and change their pharmacokinetic characteristics. Indeed, the brain-plasma ratio of CBDA in a Tween-based vehicle was about 50 times higher than that in a vegetable oil vehicle (1.9 and 0.04 respectively). CBCA may possibly be a substrate because nonionic surfactants have been demonstrated to block P-glycoprotein (P-gp)-mediated transport, and many phytocannabinoids are recognized P-gp substrates (Anderson et al. 2021).

The pharmacokinetic characteristics of CBCA, CBGA, and CBDA in mice following intraperitoneal injection as a solution dissolved in a vegetable oil carrier were only described by Lyndsey et al. (Anderson et al. 2021). With a tmax of 30 min and a brief half-life of 24 min, CBCA demonstrated quick absorption. CBCA was not detectable in brain tissue. CBGA had a slightly distinct pharmacokinetic profile, with a peak in brain concentration that came before the plasma peak and a slightly longer tmax. However, compared to plasma, the brain's Cmax and AUC values were lower. CBDA demonstrated minimal exposure, slower brain penetration (tmax of 45 min), and rapid absorption (tmax of 30 min) (Anderson et al. 2019). The findings imply that acidic cannabinoids delivered in an oil medium have low brain permeability. Their physiologically pH-negative carboxylic acid component may be caused by their physiologically pH-negative carboxylic acid component, which hinders passive blood–brain barrier penetration.

Several variables, including differences in animal species, administration routes, and vehicles, were studied to understand its pharmacokinetic profile. CBDA has demonstrated more thorough study coverage than other cannabinoids. There were no appreciable variations in the Cmax, tmax, and AUC values of the three formulations of CBD and CBDA given orally to dogs, according to (Schwark and Wakshlag 2023). When two distinct dosages of CBD and CBDA-rich hemp oil were given to Macaca fascicularis by Johns T. et al. (2023) the plants displayed higher concentrations of CBDA than CBD, confirming its potential as a beneficial cannabinoid in medicinal settings. Clarifying these metabolic issues will require additional in vitro and in vivo research (Lacerda et al. 2025).

### Acidic cannabinoids and Alzheimer’s disease

Interlinked pathological pathways drive Alzheimer’s disease (AD)—Aβ and tau protein accumulation, chronic neuroinflammation, calcium dyshomeostasis, mitochondrial dysfunction, and synaptic failure—highlighting the appeal of therapeutic agents with multi-target mechanisms. In a mouse model of AD, CBDA and THCA restored memory deficits, decreased phosphorylated tau and hippocampus Aβ, and normalized increases of Ca^++^ (D'Souza et al. 2008). This suggests a direct connection between acids and behavioral abnormalities, as well as amyloid, tau, and calcium dyshomeostasis (Kim et al. 2023). In addition, an in-silico/in-vitro/in-vivo study found that CBDA and CBGA are multi-target, disease-modifying compounds that inhibit acetyl- and butyryl-cholinesterases (AChE/BuChE), and β-secretase-1BACE-1 enzymes. These important nodes connect cholinergic failure with the production of Aβ. This suggests that AD has a genuinely polypharmacologic multi-target drug-like (MTDL) profile (Vitale et al. 2025). According to recent preclinical research, CBDA can also repair disease-linked proteome alterations in APP/PS1 mice and rescue hippocampal long-term potentiation (LTP), which speaks to synaptic failure and network restoration (Gil et al. 2025). Microglial activation, mitochondrial function, and oxidative stress are all key factors in chronic neuroinflammation in AD, and THCA's significant PPARγ agonism has been repeatedly demonstrated to have neuroprotective and anti-inflammatory effects, even though it is not AD-exclusive (Nadal et al. 2017). Cannabinoid actions on CB2-microglial signaling (neuroinflammation), modulation of Aβ/tau pathology, and downstream effects on synaptic plasticity and cellular stress—the rationale for multi-target agents in a networked disease like AD (Li et al. 2023)—are detailed in broader cannabinoid-in-AD reviews (covering acids within the class) to help map how these findings span the Aβ–tau–inflammation–mitochondria–synapse axis. The combination of the acid-focused mouse and mechanistic studies (CBDA/THCA affecting Aβ, p-tau, Ca2 +, and memory; CBDA/CBGA inhibiting AChE/BuChE/BACE-1; CBDA restoring LTP shows that acidic cannabinoids can simultaneously engage multiple, interlinked AD pathways, which is precisely the therapeutic profile that many groups are looking for.

Acidic cannabinoids—THCA, CBDA, and CBGA—have emerged as promising, non-psychoactive compounds with such polypharmacological potential. Unlike THC, THCA shows minimal CB1 receptor binding, enabling neuroprotective activity without euphoria or cognitive impairment (McPartland et al. 2017). THCA is a potent PPARγ agonist, attenuating NF-κB–mediated inflammatory cytokines, enhancing microglial β-amyloid clearance, and improving mitochondrial function (Nadal et al. 2017; Sundararajan et al. 2006). Notably, in AD-like mouse models and complementary neuronal studies, THCA and CBDA significantly improved cognitive performance, reduced hippocampal Aβ and phosphorylated tau levels, and normalized intracellular Ca (D'Souza et al. 2008)⁺ imbalances, demonstrating potential disease-modifying effects rather than symptomatic relief (Kim et al. 2023).

CBGA, the biosynthetic “mother cannabinoid” from which THCA, CBDA, and CBCA derive, exhibits antioxidant and anti-apoptotic activity, reducing mitochondrial membrane depolarization and reactive oxygen species (ROS), both implicated in early synaptic degeneration in AD (Aso and Ferrer 2014; Wang et al. 2009). It also modulates TRPV1 and TRPM8 ion channels, influencing calcium homeostasis and neuronal excitability to reduce excitotoxicity. CBDA acts on serotonin 5-HT1A receptors, potentially improving mood, neuroplasticity, and behavioral symptoms such as apathy and aggression in AD, while also inhibiting COX-2 to suppress prostaglandin-mediated neuroinflammation (Morales et al. 2017). Recent studies show CBDA and CBGA inhibit BACE-1, butyrylcholinesterase (BuChE), and acetylcholinesterase (AChE)—enzymes linked to Aβ production and cholinergic tone—supporting their role as multitarget-directed ligands (Vitale et al. 2025).

Mechanistically, THCA engages PPARγ, a nuclear receptor that dampens NF-κB–driven cytokines and supports mitochondrial programs linked to neuronal survival; this was established in neuronal systems and in vivo neurotoxicity models and remains a key rationale for testing THCA in neuroinflammatory neurodegeneration, including AD (Nadal et al. 2017; Palomares et al. 2020; Stone et al. 2020). Complementing this, “upstream” targets in the amyloid and cholinergic pathways are now implicated for CBDA and CBGA: a 2024 open-access study found both acids inhibit BACE-1, AChE, and BuChE in the low-micromolar range, an MT-DL (multi-target-directed ligand) profile that theoretically reduces Aβ formation while stabilizing cholinergic tone, two pillars of current AD pharmacology (Vitale et al. 2025). A second 2022–2023 thread places acidic cannabinoids at the level of Ca (D'Souza et al. 2008)⁺ signaling: by blocking store-operated calcium entry (SOCE) in immune cells (Faouzi et al. 2022; Suzuki et al. 2024). The acidic cannabinoids curtailed pro-inflammatory cytokine release, highly relevant because maladaptive SOCE and microglial activation amplify synaptic loss in AD; an editorial in function highlighted this as the first demonstration of SOCE inhibition by the acid forms specifically (Kozak 2022). A comparison of decarboxylation rates in secretory cavity contents vs air-dried inflorescences, showing how matrix context and handling shift the acid: neutral balance, underscoring why cold processing, encapsulation, and rapid, low-thermal extraction matter for preserving acidity ahead of CNS delivery in AD studies (Kim et al. 2024). Keeping THCA/CBDA/CBGA in their acidic state lets researchers leverage PPARγ activation, SOCE suppression, and BACE-1/ChE inhibition that are most clearly attributed to the acid forms, not their decarboxylated products. Together with broader cannabinoid reviews that map antioxidant, anti-inflammatory, and neurotrophic signaling to improved cognition, these findings tighten the case for acidic cannabinoids as polypharmacological tools in AD research pipelines (Tyrakis et al. 2024).

### Acidic cannabinoids in pain and inflammation management

Acidic cannabinoids, especially THCA and CBDA, are gaining traction in pain science because they combine anti-inflammatory, pro-resolving, and sensory-modulating actions without the psychoactive profile of THC. In inflammatory arthritis models, THCA (as the naturally occurring Δ⁹-THCA-A) reduced joint swelling, inflammatory biomarkers, synovial hyperplasia, and cartilage damage, acting via PPARγ and peripheral CB1 signaling mechanisms that overlap neatly with current anti-inflammatory and disease-modifying strategies for rheumatoid and osteoarthritis pain (Lago-Fernandez et al., 2021). These data come from a rigorous collagen-induced arthritis study and have been independently highlighted in subsequent cannabinoid–PPAR reviews, positioning THCA as a credible analgesic/anti-inflammatory lead for chronic musculoskeletal pain (Palomares et al. 2020). Beyond joint disease, acidic cannabinoids dampen immune excitability at the calcium-signaling level: a 2022 Function report showed that several acidic forms block store-operated calcium entry (SOCE) and suppress pro-inflammatory cytokine release—an upstream anti-inflammatory mechanism relevant to neuropathic and inflammatory pain sensitization (Faouzi et al. 2022; Kozak 2022). On the sensory side, the nociception-critical TRP channels are a pharmacologic foothold: structural and functional work in 2024 clarified a cannabinoid binding site on TRPA1, complementing earlier TRPV1 literature and reinforcing how phytocannabinoids can modulate mechanical/thermal hyperalgesia and oxidative-stress–linked pain pathways—targets that are active in neuropathic pain and fibromyalgia (Amawi et al. 2024).

CBDA adds complementary mechanisms that matter clinically. Modern analyses (and updated syntheses of older bench work) indicate COX-2 suppression by CBDA at low micromolar levels, consistent with prostaglandin-driven pain control while sparing COX-1—an NSAID-like effect with a potentially better GI safety profile; a 2020 review summarizes this axis and CBDA’s related nuclear-receptor signaling (Formato et al. 2020). Translationally, oral CBDA reduced orthopedic pain in a prospective study of horses with chronic osteoarthritis, improving clinical scores without rescue analgesia—an emerging, real-world signal that aligns with its anti-inflammatory mechanism and supports movement toward controlled trials in human osteoarthritis (OA) (Aragona et al. 2024; Roseti et al. 2024). For central pain states (e.g., migraine, neuropathic pain with central sensitization), it helps that THCA and CBDA are detectable in brain tissue and can act in neuronal systems; 2023 work in an AD-like model verified CNS exposure and showed normalization of Ca (D'Souza et al. 2008)⁺ homeostasis, which is mechanistically relevant to central nociceptive amplification, even though the disease model was not pain-specific (Kim et al. 2023). Pulling these threads together, the polypharmacology of acidic cannabinoids—PPARγ activation, SOCE inhibition, TRP channel modulation, and COX-2 down-tuning—maps onto key pain drivers (inflammation, immune priming, and peripheral/central sensitization) and provides a biologically coherent rationale for testing THCA/CBDA as adjuncts or alternatives to NSAIDs and neuropathic-pain agents. Contemporary reviews in musculoskeletal and peri-operative contexts echo cautious optimism, while also noting the need for dose-finding, stability-preserving formulations (to prevent decarboxylation), and high-quality RCTs before routine clinical adoption (Botea et al. 2024; Mlost et al. 2020). Table 2 illustrates a comparison of the active and acidic forms of THCA and THC, and CBDA and CBD. Raw cannabis and hemp contain natural precursors called CBDA and THCA, which transform into active forms through decarboxylation. CBD is known for its medicinal benefits, while THC is linked to euphoria and pain relief. While THC is restricted in the US, CBD and CBDA from hemp are legally recognized.

**Table 2:** Comparison of the four main cannabinoids (CBDA, CBD, THCA, THC) in terms of differences in product forms, legal status, bioavailability, psych activity, therapeutic potential, and chemical feature

Feature	CBDA	CBD	THCA	THC	References
Chemical Nature	Acidic precursor of CBD	Decarboxylated (non-acidic) form of CBDA	Acidic precursor of THC	Decarboxylated (active) form of THCA	Anderson et al. 2019; Nadal et al. 2017; Takeda et al. 2014)
Psychoactivity	Non-psychoactive	Non-psychoactive	Non-psychoactive	Psychoactive	Anderson et al. 2019; Nadal et al. 2017; Takeda et al. 2014)
Conversion	Converts to CBD with heat or aging (decarboxylation)	Formed from CBDA by heat	Converts to THC with heat (decarboxylation)	Formed from THCA by heat	Anderson et al. 2019; Nadal et al. 2017; Takeda et al. 2014)
Potential Benefits	Anti-inflammatory, anti-nausea, anti-cancer (early research)	Anxiety relief, anti-inflammatory, seizure control	Anti-inflammatory, neuroprotective, anti-emetic	Pain relief, appetite stimulation, euphoria	Anderson et al. 2019; Nadal et al. 2017; Takeda et al. 2014)
Bioavailability	Possibly higher in raw form (early research)	Lower oral bioavailability	Better in raw cannabis (limited absorption orally)	High when smoked or vaporized	Anderson et al. 2019; Nadal et al. 2017)
Common Products	Raw hemp juice, tinctures, capsules	Oils, capsules, edibles, topicals, vapes	Raw cannabis, juicing, tinctures	Edibles, oils, smokables, vapes	Anderson et al. 2019; Nadal et al. 2017)
Legal Status	Legal where hemp products are allowed	Federally legal in U.S. if hemp-derived (< 0.3% THC)	Often legal in raw form depending on jurisdiction	Controlled substance in many regions (Schedule I in U.S.)	Anderson et al. 2019; Nadal et al. 2017)

### Acidic cannabinoids and cancer

**Fig. 2:**
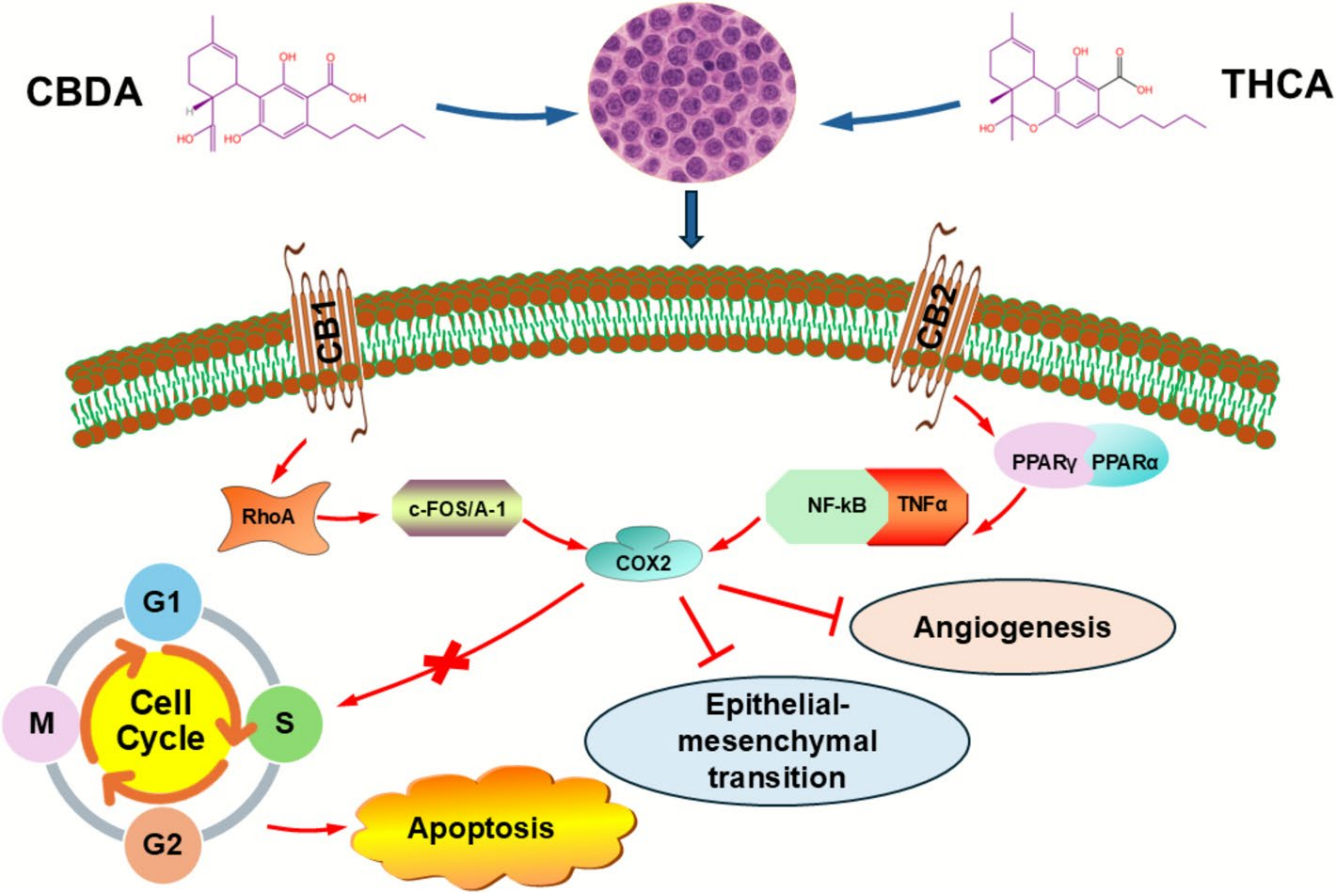
Anticancer Effects of CBDA and THCA. This figure demonstrates the mechanisms through which cannabidiolic acid (CBDA) and tetrahydrocannabinolic acid (THCA) exert their anticancer effects. Both compounds promote apoptosis, inhibit cell cycle progression, and reduce metastasis while also suppressing inflammation and engaging cannabinoid receptors. CBDA disrupts the RhoA signaling pathway by inhibiting COX-2 and reducing c-Fos/AP-1 levels, which limits tumor cell motility and promotes angiogenesis, invasion, and epithelial-mesenchymal transition (EMT). On the other hand, THCA lowers levels of TNF-α, COX-2, and pro-angiogenic mediators, while also activating peroxisome proliferator-activated receptor gamma (PPARγ) and inhibiting NF-κB and AP-1. These combined actions of THCA and CBDA shows their potential as complementary anticancer agents by targeting key hallmarks of cancer

Acidic cannabinoids are drawing oncology interest because they combine anti-metastatic, anti-inflammatory, and nuclear-receptor actions without the psychoactivity of THC, making them appeal for long-term and palliative care. The best-characterized anticancer signal remains CBDA’s anti-metastatic activity in the triple-negative breast cancer (TNBC) cell line MDA-MB-231, where CBDA downregulated COX-2, interfered with RhoA–c-fos signaling, and inhibited cell migration—mechanistic levers closely tied to epithelial-to-mesenchymal transition (EMT) and metastatic spread (Takeda et al. 2014). Minimal micromolar quantities of CBDA successfully prevented cell migration in the aggressive MDA-MB-231 TNBC model without compromising cell viability. Mechanistically, RhoA activation and cAMP-dependent protein kinase A (PKA) inhibition is connected to this anti-motility action (Takeda et al. 2012). Furthermore, CBDA exhibits a complicated interaction with PPARβ/δ signaling by downregulating COX-2 expression in MDA-MB-231 cells via pathways involving c-fos and AP-1 activity (Takeda et al. 2017). Overall, rather than directly causing cell death, CBDA seems to lessen invasive behavior by altering transcriptional programs linked to inflammation and motility (Hirao-Suzuki et al. 2020). These cell-level findings align with broader cannabinoid oncology literature showing anti-proliferative, pro-apoptotic, and anti-invasive effects across tumor types, while also highlighting that acidic forms can act outside classical CB1/CB2 pathways (Takeda et al. 2012). In parallel, THCA functions as a PPARγ agonist, a target with well-described anti-proliferative and pro-differentiation effects in cancer biology; Δ⁹-THCA’s PPARγ activity, shown initially with potent in vivo neuroprotection, supports testing THCA where tumor-promoting inflammation and metabolic rewiring are operative (Nadal et al. 2017). Contemporary reviews of medical cannabis in oncology and inflammation similarly position cannabinoids as adjuncts that may modulate tumor microenvironment tone and cancer-related symptoms, while underscoring the translational gap for acidic analogs specifically (Bodine and Kemp 2025; Mashabela and Kappo 2024). Acidic cannabinoids, specifically CBDA and THCA, are emerging as promising anticancer agents that complement or even surpass their neutral counterparts in specific mechanisms. A 2025 review of phytocannabinoids describes how these compounds inhibit tumorigenesis (Sledzinski et al. 2020). In addition to CBDA, studies have examined the effects of THCA and CBGA in cancer models, namely colorectal cancer. THCA (fraction F7) and CBGA (fraction F3)-rich unheated cannabis fractions were reported to trigger cell-cycle arrest in G_0_/G_1_ or S phases, induce apoptosis, and more efficiently reduce viability in cancer cells than in normal colon epithelial cells (Aizikovich 2020). It is noteworthy that these fractions enhanced cytotoxic and pro-apoptotic activities when combined synergistically. Modified THCA derivatives also showed enhanced anti-pancreatic cancer activity in xenograft models and in vitro, underscoring the potential of these acidic chemicals for the development of anticancer drugs. By blocking the ion channel TRPM7, which is implicated in cell migration, proliferation, and metastasis in a variety of malignancies, CBGA is connected to invasion and inflammation (Formato et al. 2020). Notably, researchers developed nitrogen-containing CBDA and THCA derivatives with enhanced anticancer activity, demonstrating in vitro and in vivo efficacy against multiple tumor cell lines, thereby overcoming the chemical instability of the acids and improving bioactivity (Aizikovich 2025). Figure 2 illustrates how CBDA and THCA interact with various cellular signaling pathways to regulate cancer cells' growth, proliferation, and metastasis.

### Acidic cannabinoids and epilepsy

Acidic cannabinoids are drawing interest as non-intoxicating antiseizure candidates because they act through mechanisms that complement (and sometimes potentiate) established therapies. Preclinical work shows CBDA enhances 5-HT1A signaling, long implicated in seizure modulation, and can be markedly more potent than CBD in serotonergic assays, which is relevant to central excitability and nausea comorbidity (Bolognini et al. 2013). In a Dravet syndrome mouse model (Scn1a^RX/+), Anderson et al. demonstrated that CBDA’s brain exposure is vehicle-dependent (very low in standard PEG/ethanol vs. markedly higher with a Tween-80 vehicle). With adequate exposure, CBDA raised the temperature threshold for generalized seizures, directly supporting anticonvulsant potential while underscoring the formulation problem for acids (Anderson et al. 2019). A 2021 Frontiers in Pharmacology review of “minor cannabinoids” synthesizes these pharmacologic threads, serotonergic modulation, ion channels, and inflammation as plausible converging routes for antiseizure activity from acidic cannabinoids, not just the neutral forms used clinically (e.g., Epidiolex) (Walsh et al. 2021).

Two recent animal studies refined how far this promise goes. In an acute rat seizure model, it was found that CBDA-enriched hemp extracts produced “entourage-like” improvements in anticonvulsant activity. CBDA appeared to boost the seizure suppression produced by other cannabinoids, while also showing standalone signals, suggesting a role as an adjunct in extract-based therapies rather than a solo magic bullet (Goerl et al. 2021). By contrast, a tested Δ⁹-THCA across multiple murine seizure models (6-Hz psychomotor, MES, and Scn1a +/− Dravet) and reported a mixed profile: THCA plus THC showed benefit in the 6-Hz model, but pure THCA alone was inconsistent and sometimes proconvulsant, highlighting model- and context-dependence and the risk of assuming uniform efficacy for acids across seizure types (Anderson et al. 2019; Benson et al. 2022). Pulling this together: the most robust 2020–2025 signals are (i) CBDA can be anticonvulsant when brain delivery is engineered correctly and may potentiate other cannabinoids; (ii) THCA requires caution because efficacy varies by dose, model, and co-administered cannabinoids; and (iii) both need stability-preserving formulations to prevent decarboxylation and achieve reproducible CNS exposure. Given the promising pharmacology but sparse human data, the field’s next step is controlled early-phase trials that lock down formulation (to keep acids acidic), exposure (PK/brain levels), and seizure endpoints—ideally in Dravet/Lennox, Gastaut, where trial infrastructure already exists—and use mechanistic biomarkers (e.g., 5-HT1A engagement) alongside clinical seizure outcomes (Anderson et al. 2019; Walsh et al. 2021).

### Use of acidic cannabinoids in medications

As of now, acidic cannabinoids such as CBDA and CBGA are not currently included in any FDA-approved or globally recognized prescription medications. They are widely sold as dietary supplements under the GRAS (Generally Recognized As Safe) label, which allows for their sale without premarket approval, if they meet 21 CFR Part 170 safety standards (Generally Recognized as Safe (GRAS) 2023). GRAS does not equate to FDA approval for medication and is regulated by the boundaries outlined in the Federal Food, Drug, and Cosmetic (FD&C) Act, §§201(ff) (Burstein 2015)(B) and 301(ll). The FDA’s drug-exclusion clauses are essential in determining the legality of acidic cannabinoids like CBDA, THCA, and CBGA. These clauses prohibit the initial marketing of licensed drugs or those undergoing extensive clinical research as foods or supplements. With a limited "first-marketed-as-food" provision, §301(ll) prohibits the addition of a drug that is permitted to normal foods. Using acidic cannabinoids in food would require either a successful GRAS demonstration or a food-additive regulation, as the FDA has not recognized any cannabinoids as a legal food ingredient through GRAS (Human Foods Program 2024) (Program HF 2024). Additionally, for an acidic cannabinoid to be considered a legal dietary ingredient, it must pass through the New Dietary Ingredient (NDI) process, which includes a 75-day premarket notification to demonstrate safety before a company can offer supplements (Human Foods Program 2025) (Program HF^2025^). While THCA contributes to the overall THC content required by federal hemp regulations, it also raises concerns regarding controlled substances (Adlin 2024). According to the FDA's more comprehensive cannabis policy stance 2023–2024, the Agency has requested that Congress develop a new framework because it has not been able to treat cannabinoids like CBD as legal foods or supplements under the law as it stands (Administration USFaD n.d.).

Despite anti-inflammatory, anticonvulsant, and anticancer properties in preclinical animals, CBDA and CBGA are chemically unstable. When heated or exposed to time, acidic cannabinoids naturally undergo decarboxylation, transforming into their more stable neutral forms, such as CBD and CBG, which are commonly found in pharmaceutical drugs like Epidiolex (U.S. FDA- approved) and Sativex (Anderson et al. 2019; Britch et al. 2021). Because they are easier to stabilize, more standardized, and have been shown to be clinically effective, neutral cannabinoids have been preferred in pharmaceutical research. Despite being sold as over-the-counter supplements, acidic cannabis formulations such as those made by Healer CBD.

 zBiomedical Pharms, and Rare cannabis company are not categorized as pharmaceuticals and do not have official regulatory approval (Biomedical Pharms, USA and Healer CBD USA) as Epidiolex. Regulations governing dietary supplements, which are less stringent than those governing prescription drugs, also apply to the marketing of these products. They are not required to go through scientific trials to prove their safety, effectiveness, or therapeutic consistency, even when they include biologically active substances like CBDA or CBGA (New Dietary Ingredient (NDI) Notification Process 2025). A lack of standardization in labeling, dosing guidelines, and purity compared to pharmaceutical products results in batch-to-batch variations in potency and cannabis concentration, as well as a lack of information on long-term safety or drug interactions (Bonn-Miller et al. 2017). The study revealed that around 70% of CBD products sold online had false labels, and more recent research keeps finding significant discrepancies from label claims and undeclared Δ9-THC contamination, demonstrating the potency/purity fluctuation (Bonn-Miller et al. 2017). Although businesses like Healer CBD prioritize transparency by focusing on physician-developed formulas and offering certificates of analysis, these measures do not replace the official validation procedures required for authorized medications (Biomedical Pharms, USA and Healer CBD, USA).

The pharmacological potential of acidic cannabinoids is, nevertheless, gaining more and more attention. Recent studies suggest that CBDA may have more bioavailability and potent anti-inflammatory properties than CBD, whereas CBGA is being investigated for its potential to treat metabolic diseases and promote neuroprotection (Benson et al. 2022; Britch et al. 2021). Acidic cannabinoids' medicinal uses, such as their potential for treating inflammatory illnesses, breast cancer, and epilepsy, are now being investigated in clinical trials. According to (Anderson et al. 2019), CBDA has demonstrated anticonvulsant effects in preclinical epileptic animals, indicating a potential future use in seizure treatment. However, before they can be used in official medical treatments, more thorough clinical trials and better techniques for stabilizing acidic cannabinoids will be required. It is projected that stabilized pharmaceutical-grade acidic cannabinoid treatments may develop when further research is conducted, providing new opportunities for cannabis-based medicine.

### Acidic cannabinoid companies

More and more businesses are incorporating acidic cannabinoids, like CBDA and CBGA, into their product development processes as their therapeutic potential continues to expand. CBDA and CBGA are appealing prospects for health and wellness improvements because, in contrast to their decarboxylated cousins, they frequently exhibit improved bioavailability, greater anti-inflammatory activity, and broader pharmacological benefits (Britch et al. 2021). Dr. Dustin Sulak launched Healer CBD, which provides a variety of formulations with a focus on acidic cannabinoids. According to their goods, bioavailability is greatly increased, and CBDA may be up to 11 times more absorbed than CBD (Healer CBD, USA). In a similar vein, Extract Labs created an Immune Support Tincture that preserves the naturally occurring acidic forms of cannabis in order to maximize its antiviral and anti-inflammatory properties (Extract Labs, USA). By using fermentation technologies to produce bio-based CBDA, biotechnology companies like Lygos, Inc. have advanced beyond plant extraction. They are providing scalable, high-purity production methods consistent with the concepts of green chemistry (Lygos, USA). By obtaining patents centered on the optimal conversion and stabilization of CBDA, Aphios Pharma LLC has aided in the development of pharmaceutical cannabinoids and highlighted the potential of this compound for future FDA-regulated therapeutic uses (Aphios Pharma, USA). The refined CBDA oil and CBGA formulations offered by Rare Cannabinoid Company, which specializes in minor and acidic cannabinoids, are frequently used with full-spectrum hemp extracts to increase the entourage effect (Rare Cannabinoid Company, USA). Similarly, by developing an immune-boosting solution that combines CBDA and CBGA with vital vitamins and minerals, CBD American Shaman took advantage of the public's enthusiasm for these cannabinoids (CBD American Shaman, USA). Adding to its line of consumer-focused goods, Northwoods Botanicals sells a Cannabinoid Mega Blend made from sustainably grown Wisconsin hemp that contains CBDA and CBGA (Northwoods Botanicals, USA).In order to address a broader range of physiological needs, Asheville Dispensary has also developed daily wellness capsules ("Resilience") that combine CBDA, CBGA, CBD, and CBG (Asheville Dispensary, USA).

Following research indicating possible antiviral effects, industrial hemp giant Hemp, Inc. announced the release of a full line of CBDA and CBGA products, including tinctures, drinks, capsules, and edibles containing raw cannabinoids (Hemp, Inc., USA). In order to optimize immune regulation, Biomedical Pharms created a CBDA: CBGA immune booster supplement that combines acidic cannabinoids with other minor cannabinoids (Biomedical Pharms, USA). In order to increase our understanding of these molecules, Nobles Corp. and the Global Cannabinoid Research Center (GCRC) are researching how acidic cannabinoids may control neurological pathways, metabolic disorders, and inflammatory diseases (Cannabis Science & Technology, USA). Since unheated cannabinoids provide synergistic benefits lost during decarboxylation, newer entrants like Rainbow Complete Spectrum are concentrating on maintaining a true "full spectrum" profile by retaining both acidic and neutral cannabinoids within their tinctures and oils (Rainbow Complete Spectrum, USA). In addition, Boulder Creek Technologies has created solventless extraction techniques that are ideal for maintaining the integrity of CBDA and CBGA, promoting cleaner and more sustainable cannabis production methods (Boulder Creek Technologies, USA). Companies in the USA that utilize acidic cannabinoids (CBDA & CBGA) are listed in Table 3. Businesses that invest in acidic cannabinoids are growing quickly, which highlights the cannabis industry's paradigm shift toward more complex, scientifically based formulas. Next-generation cannabinoid-based treatments are expected to require acidic cannabinoids.

**Table 3:** Illustrates the companies in the USA that utilize acidic cannabinoids (CBDA and CBGA)

Company	Location	Product Type	Acidic Cannabinoid	Notable Features
Healer CBD	Maine, USA	Oils drop, gummies, capsules	CBDA, CBDA	Physician developed formulas, immune support, stress relief, enhanced bioavailability
Extract Labs	Colorado, USA	Tinctures, capsules	CBDA, CBGA	Immune support tincture blending raw CBDA & CBGA
Rare Cannabinoid Company	Hawaii, USA	Tinctures, oils	CBDA, CBGA	Rare cannabinoids with 1:1:1 CBDA, CBGA, CBD blends
Biomedical Pharms	Florida, USA	Immune booster supplements	CBDA, CBGA	Formulates a CBDA: CBDGA immune booster combined with other cannabinoids
Northwoods Botanical	Wisconsin, USA	Tinctures	CBDA, CBGA	Cannabinoid mega blend tincture from organically grown hemp
Hemp, Inc	Nevada, USA	Tinctures, gummies, capsules, edibles	CBDA, CBGA	CBDA & CBGA heath studies
The Georgia Hemp Company	Georgia, USA	Tinctures, gummies, capsules, edibles, vapes, topicals	Potentially CBDA, CBGA	Full spectrum hemp extracts likely containing acidic cannabinoids
SJ Labs and Analytics	Georgia, USA	Laboratory testing services	CBDA, CBGA	Cannabinoid testing, CBDA & CBGA analysis
Lygos, Inc	California, USA	Bio-based CBDA via fermentation	CBDA	High purity CBDA sustainability using green chemistry
Aphios Pharma LLc	Massachusetts, USA	Pharmaceutical cannabinoid development	CBDA	Stabilized CBDA for future pharmaceutical use
CBD American Shaman	Kansas, USA	Immune boosting tinctures with vitamins	CBDA, CBGA	Combines CBDA & CBGA with vitamins for immune heath
Asheville Dispensary	North Carolina, USA	Daily wellness capsules with CBDA/CBGA	CBDA, CBGA	Cannabinoid blends for daily heath support
Naobles Corp./Global Cannabinoid Research Center (GCRC)	California, USA	Cannabinoid research and therapeutics exploration	CBDA, CBGA	Researching acidic cannabinoids for neurological & inflammatory disorders
Tweedle Farms	Oregon, USA	Full spectrum tinctures and oils	CBDA, CBGA	Maintains acidic cannabinoids integrity for full spectrum benefits
Boulder Creek Technologies	Colorado, USA	Solventless extracts preserving acidic cannabinoids	CBDA, CBGA	Developed solventless methods to preserve CBDA & CBGA during extractions

Leading firm ElleVet Sciences is dedicated to creating CBD and CBDA formulations for pets, particularly dogs. They have carried out peer-reviewed clinical trials that are aimed explicitly at dogs with osteoarthritis, including a randomized, placebo-controlled crossover study at Cornell University College of Veterinary Medicine (Gamble et al. 2018; White 2023; Loftus 2020). Published safety and pharmacokinetic studies that examine drug-drug interactions in canine liver microsomes and highlight the distinct metabolic pathways of CBD and CBDA were part of their study. Besides supporting ex vivo and in vitro studies that evaluate the anti-inflammatory benefits of their blended CBD/CBDA formulations using canine samples, ElleVet claims to support 15 peer-reviewed studies and numerous research and development projects (Gamble et al. 2018; White 2023; Loftus 2020).

The full-spectrum extract NTI164, created by the Australian biopharmaceutical company Neurotech International, is CBDA-dominant and is presently being researched for potential use in human medicine. Numerous clinical investigations that are subject to peer review have examined the extract. A research conducted by Keating et al. 2015 (Keating et al. 2025) described a Phase I/II open-label experiment in Rett syndrome patients, which showed notable improvements in neurological and behavioral symptoms in addition to an exceptional safety profile. Additionally, pharmacokinetic data from the trial showed that CBDA was the prominent circulating cannabinoid after the dose. The anti-inflammatory and neuroprotective properties of the same formulation were also the subject of research (Ross-Munro et al. 2024), highlighting the significance of acidic cannabinoids in the treatment of inflammatory and neurodevelopmental illnesses. Other companies may not yet have peer-reviewed publications or DEA licenses; their inclusion could serve to illustrate the broader industry trend toward innovation in acidic cannabinoid formulations.

### Limitations

Despite growing interest in acidic cannabinoids such as THCA and CBDA, several limitations hinder their translation from preclinical promise to clinical use. A major challenge is their chemical instability—both compounds readily undergo decarboxylation into their neutral forms (THC, CBD) when exposed to heat, light, or prolonged storage, which can significantly alter pharmacological activity (Urvashi et al. 2024; Kim et al. 2024). This instability complicates dosing accuracy and standardization, particularly in botanical preparations, and makes it challenging to ensure that patients are receiving the intended acidic form. Furthermore, pharmacokinetic studies indicate that CBDA and THCA generally exhibit low oral bioavailability, short half-lives, and limited central nervous system penetration under standard formulation conditions, with brain-to-plasma ratios remaining low unless specialized delivery systems (e.g., surfactants, nanoemulsions) are employed (Lacerda et al. 2025).

Due to its chemical instability, CBCA is easily changed into CBC by heat, light, or extended storage, which has an impact on measurement and retention (Tahir et al. 2021; Wang et al. 2016; Urvashi et al. 2024; Lim et al. 2021). Its limited biosynthetic pathways usually make it a modest product when compared to THCA and CBDA. Interestingly, little is known about the pharmacological effects of CBCA; most of the data comes from CBC, its neutral cousin. Achieving systematic effectiveness may need non-decarboxylating formulations, as evidenced by the variety and complexity of pharmacokinetics highlighted by the available research. Additionally, the requirement to avoid decarboxylation during analytical procedures creates quantification issues. These studies suggest that CBCA is a low-abundance, delicate substance that needs to be handled carefully and thoroughly validated for accurate research (Tahir et al. 2021; Urvashi et al. 2024; Lim et al. 2021).

Another limitation is the scarcity of high-quality human clinical data. While preclinical studies and anecdotal reports suggest potential anti-inflammatory, neuroprotective, antiemetic, and anticancer effects, the evidence base is dominated by in vitro assays and animal models, with few randomized controlled trials to validate safety, optimal dosing, and efficacy in humans (Formato et al. 2020; Walsh et al. 2021). Regulatory and manufacturing barriers also limit progress. Because acidic cannabinoids are derived from cannabis plant material, legal restrictions on cannabis cultivation and research in many jurisdictions constrain clinical investigation and large-scale production. Moreover, the lack of validated analytical methods that can accurately quantify CBDA and THCA without inducing artifactual decarboxylation during sample preparation hampers both research and quality control (Lacerda et al. 2025). Until these chemical, pharmacokinetic, and regulatory challenges are addressed through innovations in stabilization, formulation, and clinical trial design, acidic cannabinoids will remain promising but experimental therapeutic agents, with applications that are not yet ready for broad medical adoption.

## Discussion

The medicinal flexibility of acidic cannabinoids, especially THCA and CBDA, is being clarified by the growing field of cannabis research. These non-psychoactive substances, which were previously disregarded in favor of their decarboxylated counterparts, have revealed themselves to be pharmacologically active substances with unique anti-inflammatory, neuroprotective, antiemetic, and maybe anticancer qualities. They may be used as adjuncts or substitutes in medicinal formulations because of their distinct modes of action, which set them apart from CBD and THC. These mechanisms include COX-2 inhibition, 5-HT1A receptor modulation, TRP channel interaction, and PPARγ activation (Tahir et al. 2021; Gagne et al. 2012; Flores-Sanchez and Verpoorte 2008; Kim et al. 2023).

In models of pain, inflammation, and epilepsy, the pharmacological effects of CBDA are especially noteworthy. With its potent anti-inflammatory properties and specific inhibition of cyclooxygenase-2, CBDA presents a compelling non-opioid alternative for treating chronic pain. Its involvement in controlling anxiety, nausea, and seizure activity is further supported by its high affinity for serotonin 5-HT1A receptors; multiple preclinical investigations have shown its anti-hyperalgesic and anticonvulsant properties (Anderson et al. 2019; Bolognini et al. 2013; Walsh et al. 2021; Goerl et al. 2021). Further in vivo confirmation is required, although preliminary data indicates that CBDA may disrupt metastatic signaling pathways in triple-negative breast cancer cell lines, potentially by downregulating pro-tumorigenic enzymes and altering RhoA activity. Due to its capacity to function as a PPARγ agonist and its role in cytokine regulation, specifically the inhibition of TNF-α and IL-6, THCA has also demonstrated promise in several preclinical investigations (Bolognini et al. 2013; Takeda et al. 2014). These processes are the basis for its anti-inflammatory and neuroprotective properties, which are especially important in diseases like Alzheimer's, where neuroinflammation is a major pathogenic factor. Although THCA has had inconsistent outcomes in models of epilepsy, either being ineffective or even proconvulsant in specific seizure scenarios, when combined with other cannabinoids, it may increase its anticonvulsant effectiveness (Nadal et al. 2017; Kim et al. 2023; Sundararajan et al. 2006). As therapeutic approaches, whole-plant and acidic cannabinoid-rich preparations are becoming increasingly popular.

According to pharmacokinetic studies, CBDA and THCA are quickly absorbed orally; nevertheless, their short plasma half-lives and restricted CNS penetration may limit their effectiveness when taken alone. Their continuous development is supported by current formulation technology developments, including liposomal carriers and nanoemulsions, which have shown promise in improving brain bioavailability. A significant factor in the usage of these cannabinoids in treatment-resistant, geriatric, and pediatric populations is their non-psychoactive profile, which permits long-term administration with little cognitive adverse effects.

Even with the encouraging preclinical evidence, the shortage of well-monitored human clinical trials is a significant drawback. The majority of the information currently available comes from rodent models and in vitro research, which makes extrapolating results to patient populations challenging. Additionally, to maintain therapeutic effectiveness, novel extraction and storage techniques are required due to concerns about compound stability, including heat sensitivity and decarboxylation. For acidic cannabinoids to reach their full potential in mainstream medicine, the field must prioritize pharmacovigilance frameworks, clinical validation, and optimal dose trials.

## Conclusion

CBDA, THCA, and CBGA are examples of acidic cannabinoids, a distinct and exciting class of medicinal substances derived from cannabis. CBGA, produced when geranyl pyrophosphate (GPP) combines with OLA (Gulck and Moller 2020), serves as the biosynthetic precursor to all phytocannabinoids and undergoes enzymatic conversion followed by decarboxylation to yield THCA, CBDA, or CBCA (Tahir et al. 2021; Flores-Sanchez and Verpoorte 2008). The pharmacological actions of these acidic forms are varied and include the inhibition of enzymes that produce β-amyloid, the suppression of store-operated calcium entry (SOCE), strong anti-inflammatory effects, and activation of nuclear receptors like PPARγ and PPARα that are associated with metabolic regulation and neuroprotection. Both CBDA and THCA have demonstrated the ability to reduce β-amyloid and phosphorylated tau (p-tau) accumulation in hippocampal regions—effects relevant to neurodegenerative diseases like Alzheimer’s—while also providing additional therapeutic benefits through COX-2 inhibition, 5-HT1A receptor modulation, and other pathways. Importantly, they are particularly well-suited for long-term usage in sensitive groups, including children and the elderly, for illnesses ranging from cancer and epilepsy to chronic pain and inflammatory disorders, since they are non-psychoactive.

The clinical application of acidic cannabinoids is fraught with difficulties, despite their potential. Their chemical instability makes formulation, dose constancy, and pharmaceutical standardization more difficult since it causes fast decarboxylation when heated or stored for an extended period. Furthermore, their use in illnesses of the central nervous system is limited by their low bioavailability and limited brain penetration in unaltered forms; however, new delivery methods, including lipid-based carriers, nanoemulsions, and other sophisticated formulations, provide encouraging alternatives. The absence of strong human clinical studies continues to be a significant deficiency, resulting in unanswered issues regarding safety, optimal dosing, effectiveness, and interactions across drugs. This is made more difficult by the analytical challenge of distinguishing between acidic and neutral cannabis in pharmacokinetic and pharmacodynamic studies, underscoring the need for more advanced detection methods. Nevertheless, with the growing scientific interest, evolving cannabis regulations, and increasing public demand for whole-plant, full-spectrum therapeutics, THCA and CBDA are well-positioned to become integral components of precision cannabinoid medicine. Continued research, innovative formulation technologies, and supportive policy changes could solidify their role in bridging the gap between targeted pharmacology and natural therapeutic approaches.

## Data Availability

This article includes all data generated and analyzed in the study.

## References

[CR1] Adlin B. DEA says ‘THCA does not meet the definition’ of legal hemp as Congress weighs cannabinoid recriminalization in Farm Bill. Marijuana Moment. 2024.

[CR2] Administration USFaD. FDA Regulation of Cannabis and Cannabis-Derived Products: Q&A 2024. n.d. Available from: https://www.fda.gov/news-events/public-health-focus/fda-regulation-cannabis-and-cannabis-derived-products-including-cannabidiol-cbd.

[CR3] Aizikovich A. Anticancer effect of new cannabinoids derived from tetrahydrocannabinolic acid on PANC-1 and AsPC-1 human pancreas tumor cells. J Pancreat Cancer. 2020;6(1):40–4. 32642629 10.1089/pancan.2020.0003 PMC7337241

[CR4] Aizikovich A. Cannabinolic acids derivatives as a new anticancer drugs. Med Res Arch. 2025;13(6).

[CR5] Amaral Silva D, Pate DW, Clark RD, Davies NM, El-Kadi AOS, Lobenberg R. Phytocannabinoid drug-drug interactions and their clinical implications. Pharmacol Ther. 2020;215:107621. 32615127 10.1016/j.pharmthera.2020.107621

[CR6] Amawi T, Nmarneh A, Noy G, Ghantous M, Niv MY, Di Pizio A, et al. Identification of the TRPA1 cannabinoid-binding site. Pharmacol Res. 2024;209:107444. 39368566 10.1016/j.phrs.2024.107444

[CR7] Anderson LL, Low IK, Banister SD, McGregor IS, Arnold JC. Pharmacokinetics of phytocannabinoid acids and anticonvulsant effect of cannabidiolic acid in a mouse model of Dravet syndrome. J Nat Prod. 2019;82(11):3047–55. 31686510 10.1021/acs.jnatprod.9b00600

[CR8] Anderson LL, Ametovski A, Lin Luo J, Everett-Morgan D, McGregor IS, Banister SD, et al. Cannabichromene, Related Phytocannabinoids, and 5-Fluoro-cannabichromene Have Anticonvulsant Properties in a Mouse Model of Dravet Syndrome. ACS Chem Neurosci. 2021;12(2):330–9. 33395525 10.1021/acschemneuro.0c00677

[CR9] Aragona F, Tabbi M, Gugliandolo E, Giannetto C, D’Angelo F, Fazio F, et al. Role of cannabidiolic acid or the combination of cannabigerol/cannabidiol in pain modulation and welfare improvement in horses with chronic osteoarthritis. Front Vet Sci. 2024;11:1496473. 39720409 10.3389/fvets.2024.1496473 PMC11668182

[CR10] Aso E, Ferrer I. Cannabinoids for treatment of Alzheimer’s disease: moving toward the clinic. Front Pharmacol. 2014;5:37. 24634659 10.3389/fphar.2014.00037 PMC3942876

[CR11] Benson MJ, Anderson LL, Low IK, Luo JL, Kevin RC, Zhou C, et al. Evaluation of the Possible Anticonvulsant Effect of Delta(9)-Tetrahydrocannabinolic Acid in Murine Seizure Models. Cannabis Cannabinoid Res. 2022;7(1):46–57. 33998858 10.1089/can.2020.0073 PMC8864425

[CR12] Bodine M, Kemp AK. Medical Cannabis Use in Oncology. StatPearls. Treasure Island (FL); 2025. 34283433

[CR13] Bolognini D, Rock EM, Cluny NL, Cascio MG, Limebeer CL, Duncan M, et al. Cannabidiolic acid prevents vomiting in *Suncus murinus* and nausea-induced behaviour in rats by enhancing 5-HT1A receptor activation. Br J Pharmacol. 2013;168(6):1456–70. 23121618 10.1111/bph.12043 PMC3596650

[CR14] Bonn-Miller MO, Loflin MJE, Thomas BF, Marcu JP, Hyke T, Vandrey R. Labeling accuracy of cannabidiol extracts sold online. JAMA. 2017;318(17):1708–9. 29114823 10.1001/jama.2017.11909 PMC5818782

[CR15] Borowicz-Reutt K, Czernia J, Krawczyk M. CBD in the treatment of epilepsy. Molecules. 2024;29(9). 10.3390/molecules29091981 PMC11085483 38731471

[CR16] Botea MO, Andereggen L, Urman RD, Luedi MM, Romero CS. Cannabinoids for acute pain management: approaches and rationale. Curr Pain Headache Rep. 2024;28(7):681–9. 38607548 10.1007/s11916-024-01252-4 PMC11271357

[CR17] Britch SC, Babalonis S, Walsh SL. Cannabidiol: pharmacology and therapeutic targets. Psychopharmacology. 2021;238(1):9–28. 33221931 10.1007/s00213-020-05712-8 PMC7796924

[CR18] Burstein S. Cannabidiol (CBD) and its analogs: a review of their effects on inflammation. Bioorg Med Chem. 2015;23(7):1377–85. 25703248 10.1016/j.bmc.2015.01.059

[CR19] Castillo-Arellano J, Canseco-Alba A, Cutler SJ, Leon F. The polypharmacological effects of cannabidiol. Molecules. 2023;28(7). 10.3390/molecules28073271 PMC10096752 37050032

[CR20] Chayasirisobhon S. Mechanisms of action and pharmacokinetics of cannabis. Perm J. 2020;25:1–3. 33635755 10.7812/TPP/19.200 PMC8803256

[CR21] Citti C, Linciano P, Russo F, Luongo L, Iannotta M, Maione S, et al. A novel phytocannabinoid isolated from Cannabis sativa L. with an in vivo cannabimimetic activity higher than Delta(9)-tetrahydrocannabinol: Delta(9)-Tetrahydrocannabiphorol. Sci Rep. 2019;9(1):20335. 31889124 10.1038/s41598-019-56785-1 PMC6937300

[CR22] Colvin EK, Hudson AL, Anderson LL, Kumar RP, McGregor IS, Howell VM, et al. An Examination of the Anti-Cancer Properties of Plant Cannabinoids in Preclinical Models of Mesothelioma. Cancers (Basel). 2022;14(15). 10.3390/cancers14153813 PMC9367527 35954477

[CR23] De Petrocellis L, Di Marzo V. Non-CB1, non-CB2 receptors for endocannabinoids, plant cannabinoids, and synthetic cannabimimetics: focus on G-protein-coupled receptors and transient receptor potential channels. J Neuroimmune Pharmacol. 2010;5(1):103–21. 19847654 10.1007/s11481-009-9177-z

[CR24] D’Souza DC, Ranganathan M, Braley G, Gueorguieva R, Zimolo Z, Cooper T, et al. Blunted psychotomimetic and amnestic effects of delta-9-tetrahydrocannabinol in frequent users of cannabis. Neuropsychopharmacology. 2008;33(10):2505–16. 18185500 10.1038/sj.npp.1301643 PMC3799954

[CR25] Etchart MG, Anderson LL, Ametovski A, Jones PM, George AM, Banister SD, et al. In vitro evaluation of the interaction of the cannabis constituents cannabichromene and cannabichromenic acid with ABCG2 and ABCB1 transporters. Eur J Pharmacol. 2022;922:174836. 35306000 10.1016/j.ejphar.2022.174836

[CR26] Faouzi M, Wakano C, Monteilh-Zoller MK, Neupane RP, Starkus JG, Neupane JB, et al. Acidic cannabinoids suppress proinflammatory cytokine release by blocking store-operated calcium entry. Function. 2022;3(4):zqac033. 35910331 10.1093/function/zqac033 PMC9334010

[CR27] Flores-Sanchez IJ, Verpoorte R. PKS activities and biosynthesis of cannabinoids and flavonoids in *Cannabis sativa* L. plants. Plant Cell Physiol. 2008;49(12):1767–82. 18854334 10.1093/pcp/pcn150

[CR28] Formato M, Crescente G, Scognamiglio M, Fiorentino A, Pecoraro MT, Piccolella S, et al. (‒)-Cannabidiolic acid, a still overlooked bioactive compound: an introductory review and preliminary research. Molecules. 2020;25(11). 10.3390/molecules25112638 PMC7321064 32517131

[CR29] Fulvio F, Mandolino G, Citti C, Pecchioni N, Cannazza G, Paris R. Phytocannabinoids biosynthesis during early stages of development of young *Cannabis sativa* L. seedlings: integrating biochemical and transcription data. Phytochemistry. 2023;214:113793. 37479208 10.1016/j.phytochem.2023.113793

[CR30] Gagne SJ, Stout JM, Liu E, Boubakir Z, Clark SM, Page JE. Identification of olivetolic acid cyclase from *Cannabis sativa* reveals a unique catalytic route to plant polyketides. Proc Natl Acad Sci U S A. 2012;109(31):12811–6. 22802619 10.1073/pnas.1200330109 PMC3411943

[CR31] Gamage TF, Lichtman AH. The endocannabinoid system: role in energy regulation. Pediatr Blood Cancer. 2012;58(1):144–8. 22076835 10.1002/pbc.23367 PMC3696506

[CR32] Gamble LJ, Boesch JM, Frye CW, Schwark WS, Mann S, Wolfe L, et al. Pharmacokinetics, safety, and clinical efficacy of cannabidiol treatment in osteoarthritic dogs. Front Vet Sci. 2018;5:165. 30083539 10.3389/fvets.2018.00165 PMC6065210

[CR33] Generally Recognized as Safe (GRAS). 2023.

[CR34] Gil B, Sullivan M, Scaife C, Glennon JC, Herron C. Cannabidiolic Acid Rescues Deficits in Hippocampal Long-Term Potentiation in Models of Alzheimer's Disease: An Electrophysiological and Proteomic Analysis. Int J Mol Sci. 2025;26(10). 10.3390/ijms26104944 PMC12112199 40430085

[CR35] Goerl B, Watkins S, Metcalf C, Smith M, Beenhakker M. Cannabidiolic acid exhibits entourage-like improvements of anticonvulsant activity in an acute rat model of seizures. Epilepsy Res. 2021;169:106525. 33310415 10.1016/j.eplepsyres.2020.106525 PMC7855831

[CR36] Gulck T, Moller BL. Phytocannabinoids: origins and biosynthesis. Trends Plant Sci. 2020;25(10):985–1004. 32646718 10.1016/j.tplants.2020.05.005

[CR37] Haney M, Vallee M, Fabre S, Collins Reed S, Zanese M, Campistron G, et al. Signaling-specific inhibition of the CB(1) receptor for cannabis use disorder: phase 1 and phase 2a randomized trials. Nat Med. 2023;29(6):1487–99. 37291212 10.1038/s41591-023-02381-w PMC10287566

[CR38] Hirao-Suzuki M, Takeda S, Koga T, Takiguchi M, Toda A. Cannabidiolic acid dampens the expression of cyclooxygenase-2 in MDA-MB-231 breast cancer cells: possible implication of the peroxisome proliferator-activated receptor beta/delta abrogation. J Toxicol Sci. 2020;45(4):227–36. 32238697 10.2131/jts.45.227

[CR39] Johns TN, Wakshlag JJ, Lyubimov AV, Zakharov A, Burnside WM. Pharmacokinetics of cannabidiol-/cannabidiolic acid-rich hemp oil in juvenile cynomolgus macaques (Macaca fascicularis). Front Vet Sci. 2023;10:1286158. 10.3389/fvets.2023.1286158 PMC10716325 38094499

[CR40] Keating BA, Ogru Y, Duthy TG, Douglas L, Lichkus K, Isikgel E, et al. Full-spectrum medicinal cannabis plant extract 0.08% THC (NTI164) Improves Symptoms of Rett Syndrome: An Open-Label Study. J Paediatr Child Health. 2025. 10.1111/jpc.70122 40568811

[CR41] Khouchlaa A, Khouri S, Hajib A, Zeouk I, Amalich S, Msairi S, et al. Health benefits, pharmacological properties, and metabolism of cannabinol: a comprehensive review. Ind Crops Prod. 2024;213:118359.

[CR42] Kim ES, Park SH, Kinney CA, Olejar KJ, Corredor-Perilla IC. Comparison of decarboxylation rates of acidic cannabinoids between secretory cavity contents and air-dried inflorescence extracts in *Cannabis sativa* cv. “Cherry Wine.” Sci Rep. 2024;14(1):16411. 39013926 10.1038/s41598-024-66420-3 PMC11252385

[CR43] Kim J, Choi P, Park YT, Kim T, Ham J, Kim JC. The Cannabinoids, CBDA and THCA, Rescue Memory Deficits and Reduce Amyloid-Beta and Tau Pathology in an Alzheimer's Disease-like Mouse Model. Int J Mol Sci. 2023;24(7). 10.3390/ijms24076827 PMC10095267 37047798

[CR44] Kovalchuk O, Kovalchuk I. Cannabinoids as anticancer therapeutic agents. Cell Cycle. 2020;19(9):961–89. 32249682 10.1080/15384101.2020.1742952 PMC7217364

[CR45] Kozak JA. Suppression of store-operated calcium entry channels and cytokine release by cannabinoids. Function. 2022;3(5):zqac044. 36168590 10.1093/function/zqac044 PMC9508850

[CR46] Krzyzewska A, Kloza M, Kozlowska H. Comprehensive mini-review: therapeutic potential of cannabigerol - focus on the cardiovascular system. Front Pharmacol. 2025;16:1561385. 40206058 10.3389/fphar.2025.1561385 PMC11979378

[CR47] Lacerda M, Carona A, Castanheira S, Falcao A, Bicker J, Fortuna A. Pharmacokinetics of Non-Psychotropic Phytocannabinoids. Pharmaceutics. 2025;17(2). 10.3390/pharmaceutics17020236 PMC11858989 40006604

[CR48] Lago-Fernandez A, Zarzo-Arias S, Jagerovic N, Morales P. Relevance of Peroxisome Proliferator Activated Receptors in Multitarget Paradigm Associated with the Endocannabinoid System. Int J Mol Sci. 2021;22(3). 10.3390/ijms22031001 PMC7863932 33498245

[CR49] Li S, Huang Y, Yu L, Ji X, Wu J. Impact of the cannabinoid system in Alzheimer’s disease. Curr Neuropharmacol. 2023;21(3):715–26. 35105293 10.2174/1570159X20666220201091006 PMC10207907

[CR50] Li S, Li W, Malhi NK, Huang J, Li Q, Zhou Z, et al. Cannabigerol (CBG): a comprehensive review of its molecular mechanisms and therapeutic potential. Molecules. 2024;29(22). 10.3390/molecules29225471 PMC11597810 39598860

[CR51] Lim KJH, Lim YP, Hartono YD, Go MK, Fan H, Yew WS. Biosynthesis of nature-inspired unnatural cannabinoids. Molecules. 2021;26(10). 10.3390/molecules26102914 PMC8156804 34068935

[CR52] Loftus J. Ex vivo & in vitro anti-inflammatory effects of a mixed CBD/CBDA hemp oil formulation. 2020.

[CR53] Luo X, Reiter MA, d’Espaux L, Wong J, Denby CM, Lechner A, et al. Complete biosynthesis of cannabinoids and their unnatural analogues in yeast. Nature. 2019;567(7746):123–6. 30814733 10.1038/s41586-019-0978-9

[CR54] Martinez Naya N, Kelly J, Corna G, Golino M, Abbate A, Toldo S. Molecular and cellular mechanisms of action of cannabidiol. Molecules. 2023;28(16). 10.3390/molecules28165980 PMC10458707 37630232

[CR55] Mashabela MD, Kappo AP. Anti-cancer and anti-proliferative potential of cannabidiol: a cellular and molecular perspective. Int J Mol Sci. 2024;25(11). 10.3390/ijms25115659 PMC11171526 38891847

[CR56] McPartland JM, Duncan M, Di Marzo V, Pertwee RG. Are cannabidiol and Delta(9) -tetrahydrocannabivarin negative modulators of the endocannabinoid system? A Systematic Review. Br J Pharmacol. 2015;172(3):737–53. 25257544 10.1111/bph.12944 PMC4301686

[CR57] McPartland JM, MacDonald C, Young M, Grant PS, Furkert DP, Glass M. Affinity and efficacy studies of tetrahydrocannabinolic acid A at cannabinoid receptor types one and two. Cannabis Cannabinoid Res. 2017;2(1):87–95. 28861508 10.1089/can.2016.0032 PMC5510775

[CR58] Meanti R, Bresciani E, Rizzi L, Molteni L, Coco S, Omeljaniuk RJ, et al. Cannabinoid receptor 2 (CB2R) as potential target for the pharmacological treatment of neurodegenerative diseases. Biomed Pharmacother. 2025;186:118044. 40209306 10.1016/j.biopha.2025.118044

[CR59] Mlost J, Bryk M, Starowicz K. cannabidiol for pain treatment: focus on pharmacology and mechanism of action. Int J Mol Sci. 2020;21(22). 10.3390/ijms21228870 PMC7700528 33238607

[CR60] Morales P, Reggio PH, Jagerovic N. An overview on medicinal chemistry of synthetic and natural derivatives of cannabidiol. Front Pharmacol. 2017;8:422. 28701957 10.3389/fphar.2017.00422 PMC5487438

[CR61] Morimoto S, Komatsu K, Taura F, Shoyama Y. Purification and characterization of cannabichromenic acid synthase from Cannabis sativa. Phytochemistry. 1998;49(6):1525–9. 9862135 10.1016/s0031-9422(98)00278-7

[CR62] Nadal X, Del Rio C, Casano S, Palomares B, Ferreiro-Vera C, Navarrete C, et al. Tetrahydrocannabinolic acid is a potent PPARgamma agonist with neuroprotective activity. Br J Pharmacol. 2017;174(23):4263–76. 28853159 10.1111/bph.14019 PMC5731255

[CR63] Nallathambi R, Mazuz M, Ion A, Selvaraj G, Weininger S, Fridlender M, et al. Anti-Inflammatory Activity in Colon Models Is Derived from Delta9-Tetrahydrocannabinolic Acid That Interacts with Additional Compounds in Cannabis Extracts. Cannabis Cannabinoid Res. 2017;2(1):167–82. 29082314 10.1089/can.2017.0027 PMC5627671

[CR64] Navarro G, Varani K, Reyes-Resina I, Sanchez de Medina V, Rivas-Santisteban R, Sanchez-Carnerero Callado C, et al. Cannabigerol Action at Cannabinoid CB(1) and CB(2) Receptors and at CB(1)-CB(2) Heteroreceptor Complexes. Front Pharmacol. 2018;9:632. 29977202 10.3389/fphar.2018.00632 PMC6021502

[CR65] New Dietary Ingredient (NDI) Notification Process. U.S. Food and Drug Administration; 2025.

[CR66] Palomares O. Could we co-opt the cannabinoid system for asthma therapy? Expert Rev Clin Immunol. 2023;19(10):1183–6. 37420178 10.1080/1744666X.2023.2235082

[CR67] Palomares B, Garrido-Rodriguez M, Gonzalo-Consuegra C, Gomez-Canas M, Saen-Oon S, Soliva R, et al. Delta(9) -Tetrahydrocannabinolic acid alleviates collagen-induced arthritis: Role of PPARgamma and CB(1) receptors. Br J Pharmacol. 2020;177(17):4034–54. 32510591 10.1111/bph.15155 PMC7429492

[CR68] Park SH, Pauli CS, Gostin EL, Staples SK, Seifried D, Kinney C, et al. Effects of short-term environmental stresses on the onset of cannabinoid production in young immature flowers of industrial hemp (*Cannabis sativa* L.). J Cannabis Res. 2022;4(1):1. 34980266 10.1186/s42238-021-00111-y PMC8725245

[CR69] Perrotin-Brunel H, Kroon M, Roosmalen M, Spronsen J, Peters C, Witkamp G. Solubility of non-psychoactive cannabinoids in supercritical carbon dioxide and comparison with psychoactive cannabinoids. J Supercrit Fluids. 2010;55(2):603–8.

[CR70] Pertwee RG. The diverse CB1 and CB2 receptor pharmacology of three plant cannabinoids: delta9-tetrahydrocannabinol, cannabidiol and delta9-tetrahydrocannabivarin. Br J Pharmacol. 2008;153(2):199–215. 17828291 10.1038/sj.bjp.0707442 PMC2219532

[CR71] Program HF. Three GRAS Notices for Hemp Seed-Derived Ingredients for Food. U.S. Food and Drug Administration; 2024.

[CR72] Program HF. New Dietary Ingredient (NDI) Notification Process. U.S. Food and Drug Administration; 2025.

[CR73] Qi M, Liu T, Zhang W, Wan H, Wang M, Kang W, et al. Enhancing cannabichromenic acid biosynthesis in *Saccharomyces cerevisiae*. ACS Synth Biol. 2025;14(2):531–41. 39808700 10.1021/acssynbio.4c00721

[CR74] Reichel P, Munz S, Hartung J, Kotiranta S, Graeff-Honninger S. Impacts of Different Light Spectra on CBD, CBDA and Terpene Concentrations in Relation to the Flower Positions of Different Cannabis Sativa L. Strains. Plants (Basel). 2022;11(20). 10.3390/plants11202695 PMC9612076 36297719

[CR75] Rock EM, Kopstick RL, Limebeer CL, Parker LA. Tetrahydrocannabinolic acid reduces nausea-induced conditioned gaping in rats and vomiting in *Suncus murinus*. Br J Pharmacol. 2013;170(3):641–8. 23889598 10.1111/bph.12316 PMC3792001

[CR76] Roseti L, Borciani G, Amore E, Grigolo B. Cannabinoids in the inflamed synovium can be a target for the treatment of rheumatic diseases. Int J Mol Sci. 2024;25(17). 10.3390/ijms25179356 PMC11394920 39273304

[CR77] Ross-Munro E, Isikgel E, Fleiss B. Evaluation of the efficacy of a full-spectrum low-THC cannabis plant extract using in vitro models of inflammation and excitotoxicity. Biomolecules. 2024;14(11). 10.3390/biom14111434 PMC11592195 39595610

[CR78] Russo EB. Taming THC: potential cannabis synergy and phytocannabinoid-terpenoid entourage effects. Br J Pharmacol. 2011;163(7):1344–64. 21749363 10.1111/j.1476-5381.2011.01238.x PMC3165946

[CR79] Schwark WS, Wakshlag JJ. A One Health perspective on comparative cannabidiol and cannabidiolic acid pharmacokinetics and biotransformation in humans and domestic animals. Am J Vet Res. 2023;84(5). 10.2460/ajvr.23.02.0031 36972696

[CR80] Sepulveda DE, Vrana KE, Kellogg JJ, Bisanz JE, Desai D, Graziane NM, et al. The potential of cannabichromene (CBC) as a therapeutic agent. J Pharmacol Exp Ther. 2024;391(2):206–13. 38777605 10.1124/jpet.124.002166 PMC11493452

[CR81] Sledzinski P, Nowak-Terpilowska A, Zeyland J. Cannabinoids in medicine: cancer, immunity, and microbial diseases. Int J Mol Sci. 2020;22(1). 10.3390/ijms22010263 PMC7795897 33383838

[CR82] Stone NL, Murphy AJ, England TJ, O’Sullivan SE. A systematic review of minor phytocannabinoids with promising neuroprotective potential. Br J Pharmacol. 2020;177(19):4330–52. 32608035 10.1111/bph.15185 PMC7484504

[CR83] Storozhuk MV. Cannabidiol: potential in treatment of neurological diseases, flax as a possible natural source of cannabidiol. Front Cell Neurosci. 2023;17:1131653. 37138768 10.3389/fncel.2023.1131653 PMC10150377

[CR84] Sundararajan S, Jiang Q, Heneka M, Landreth G. Ppargamma as a therapeutic target in central nervous system diseases. Neurochem Int. 2006;49(2):136–44. 16766086 10.1016/j.neuint.2006.03.020

[CR85] Suzuki S, Fleig A, Penner R. CBGA ameliorates inflammation and fibrosis in nephropathy. Sci Rep. 2023;13(1):6341. 37072467 10.1038/s41598-023-33507-2 PMC10113213

[CR86] Suzuki S, Wakano C, Monteilh-Zoller MK, Cullen AJ, Fleig A, Penner R. Cannabigerolic acid (CBGA) inhibits the TRPM7 ion channel through its kinase domain. Function. 2024;5(1):zqad069. 38162115 10.1093/function/zqad069 PMC10757070

[CR87] Tahir MN, Raz FS, Rondeau-Gagne S, Trant JF. The biosynthesis of the cannabinoids. J Cannabis Res. 2021;3(1):7. 33722296 10.1186/s42238-021-00062-4 PMC7962319

[CR88] Takeda S, Misawa K, Yamamoto I, Watanabe K. Cannabidiolic acid as a selective cyclooxygenase-2 inhibitory component in cannabis. Drug Metab Dispos. 2008;36(9):1917–21. 18556441 10.1124/dmd.108.020909

[CR89] Takeda S, Okajima S, Miyoshi H, Yoshida K, Okamoto Y, Okada T, et al. Cannabidiolic acid, a major cannabinoid in fiber-type cannabis, is an inhibitor of MDA-MB-231 breast cancer cell migration. Toxicol Lett. 2012;214(3):314–9. 22963825 10.1016/j.toxlet.2012.08.029 PMC4009504

[CR90] Takeda S, Okazaki H, Ikeda E, Abe S, Yoshioka Y, Watanabe K, et al. Down-regulation of cyclooxygenase-2 (COX-2) by cannabidiolic acid in human breast cancer cells. J Toxicol Sci. 2014;39(5):711–6. 25242400 10.2131/jts.39.711

[CR91] Takeda S, Himeno T, Kakizoe K, Okazaki H, Okada T, Watanabe K, et al. Cannabidiolic acid-mediated selective down-regulation of c-fos in highly aggressive breast cancer MDA-MB-231 cells: possible involvement of its down-regulation in the abrogation of aggressiveness. J Nat Med. 2017;71(1):286–91. 27530354 10.1007/s11418-016-1030-0

[CR92] Thomas F, Kayser O. Improving CBCA synthase activity through rational protein design. J Biotechnol. 2023;363:40–9. 36681096 10.1016/j.jbiotec.2023.01.004

[CR93] Thomas F, Schmidt C, Kayser O. Bioengineering studies and pathway modeling of the heterologous biosynthesis of tetrahydrocannabinolic acid in yeast. Appl Microbiol Biotechnol. 2020;104(22):9551–63. 33043390 10.1007/s00253-020-10798-3 PMC7595985

[CR94] Tusl J. Fluoride ion activity electrode as a suitable means for exact direct determination of urinary fluoride. Anal Chem. 1972;44(9):1693–4. 5083862 10.1021/ac60317a046

[CR95] Tyrakis P, Agridi C, Kourti M. A comprehensive exploration of the multifaceted neuroprotective role of cannabinoids in Alzheimer's disease across a decade of research. Int J Mol Sci. 2024;25(16). 10.3390/ijms25168630 PMC11354546 39201317

[CR96] Urvashi, Han JH, Hong M, Kwon TH, Druelinger M, Park SH, et al. Thermo-chemical conversion kinetics of cannabinoid acids in hemp (Cannabis sativa L.) using pressurized liquid extraction. J Cannabis Res. 2024;6(1):33. 10.1186/s42238-024-00243-x PMC11290075 39080738

[CR97] Vitale RM, Morace AM, D’Errico A, Ricciardi F, Fusco A, Boccella S, et al. Identification of Cannabidiolic and Cannabigerolic Acids as MTDL AChE, BuChE, and BACE-1 Inhibitors Against Alzheimer’s Disease by In Silico, In Vitro, and In Vivo Studies. Phytother Res. 2025;39(1):233–45. 39510547 10.1002/ptr.8369 PMC11745148

[CR98] Walsh KB, McKinney AE, Holmes AE. Minor cannabinoids: biosynthesis, molecular pharmacology and potential therapeutic uses. Front Pharmacol. 2021;12:777804. 34916950 10.3389/fphar.2021.777804 PMC8669157

[CR99] Wang X, Su B, Lee HG, Li X, Perry G, Smith MA, et al. Impaired balance of mitochondrial fission and fusion in Alzheimer’s disease. J Neurosci. 2009;29(28):9090–103. 19605646 10.1523/JNEUROSCI.1357-09.2009 PMC2735241

[CR100] Wang M, Wang YH, Avula B, Radwan MM, Wanas AS, van Antwerp J, et al. Decarboxylation Study of Acidic Cannabinoids: A Novel Approach Using Ultra-High-Performance Supercritical Fluid Chromatography/Photodiode Array-Mass Spectrometry. Cannabis Cannabinoid Res. 2016;1(1):262–71. 28861498 10.1089/can.2016.0020 PMC5549281

[CR101] White S. breakthrough study shows safety of CBD+CBDA regarding drug interactions in dogs. ElleVet Sciences; 2023.

